# Managers’ Experiences in Implementing Internet Plus Nursing Service in China: A Qualitative Study

**DOI:** 10.1155/jonm/4123747

**Published:** 2026-10-12

**Authors:** Zhifang Ren, Shuojin Fu, Shuai Jin, Yanling Wang, Qian Xiao

**Affiliations:** ^1^ School of Nursing Capital Medical University Beijing China ccmu.edu.cn; ^2^ School of Nursing Tianjin University of Traditional Chinese Medicine Tianjin China tjutcm.edu.cn; ^3^ School of Nursing Tung Wah College Hong Kong China twc.edu.hk

**Keywords:** health services accessibility, nursing administration research, nursing services, online-to-offline services, qualitative research

## Abstract

**Aim:**

To describe the experiences of managers directly involved in O2O‐style Internet Plus Nursing Service (IPNS) in China.

**Methods:**

A descriptive qualitative study design was used. Semistructured interviews were conducted with 24 participants, including 18 hospital‐based nurse managers and 6 platform managers, using maximum variation purposive sampling.

**Results:**

Four main themes were identified: (1) extending care and professional value beyond hospital walls (optimizing the delivery of continuity of care and elevating nurses’ professional fulfillment and growth drive); (2) mobilizing nursing resources amid workforce constraints (underutilization of service potential, divergent views on nurse turnover, and difficulty in utilizing community nursing resources); (3) managing service delivery across organizational boundaries (difficulty in launching the service, lack of support and assurance, and weak and challenging service regulation); and (4) building conditions for sustainable development (refining rules and division of labor, increasing publicity, and developing sustainable market‐oriented mechanisms).

**Conclusion:**

The study highlights both the benefits and challenges experienced by managers involved in O2O‐style IPNS. Sustainable development of this model requires clearer regulatory boundaries, stronger institutional support, improved platform–hospital coordination, and greater recognition of nurses’ professional roles beyond hospital settings.

**Implications for Nursing Management:**

Nursing managers should strengthen workforce planning through flexible staffing, targeted training, and appropriate incentive mechanisms. Health authorities, hospitals, and service platforms should jointly clarify responsibilities, standardize quality monitoring and documentation procedures, and provide regulatory guidance to support the safe and scalable development of O2O‐style IPNS.

## 1. Introduction

As populations age and the number of patients with chronic diseases increases, traditional nursing services face immense pressure in resource allocation and accessibility [1–3]. *Internet Plus Nursing Service (IPNS)* is a novel service model that integrates information technology with traditional nursing care. In this model, patients make payments and requests *online*, while institution‐registered nurses provide services in patients’ homes *offline*, representing a typical online‐to‐offline *(O2O)* model [4, 5]. The goal of this model is to leverage information technology to streamline the nursing process and extend the reach of nursing services, addressing the growing demand for care and promoting health equity [6, 7]. IPNS addresses healthcare limitations in traditional nursing services by optimizing nursing resource allocation through internet technology, enabling seamless access and improving service efficiency and accessibility [8, 9]. In addition, this model reduces medical costs and limits hospital visits, especially benefiting patients with mobility limitations [10]. In sum, O2O‐style IPNS offers significant benefits in enhancing healthcare efficiency and improving patient experiences; however, successful implementation requires the leadership and coordination of nurse managers.

While O2O‐style IPNS is beneficial and continues to expand in scope, its practical implementation involves ongoing institutional and operational challenges. In China, many hospitals remain cautious about implementing O2O‐style IPNS because this model extends nursing services beyond hospital settings, raising concerns about nurse safety, medical liability, service quality control, regulatory responsibility, and financial sustainability. First, policy and regulatory frameworks remain underdeveloped, lacking unified industry standards and oversight mechanisms, which makes it difficult to address issues arising during service provision effectively [11]. Additionally, nurses’ safety is a primary concern, especially given the nature of providing offline, in‐person services in patients’ homes offline, which exposes nurses to both personal and professional risks [12]. The diversity of patients’ home environments and the lack of tools further complicate effective patient safety monitoring [13]. Furthermore, safeguarding nurses’ rest time is challenging, as the time spent in traffic and in patients’ homes often extends beyond regular working hours. This increases their workload and impacts their well‐being, which, in turn, reduces their service motivation [14]. Lastly, insufficient promotion and low public awareness significantly limit service adoption; many patients and their families are unfamiliar with this model, resulting in low utilization and unrealized potential [15]. Collectively, these challenges not only undermine the sustainability of service delivery but also place increasing pressure on nursing managers, who must oversee operations, coordinate multidisciplinary inputs, and ensure quality and safety under constrained institutional conditions [16]. To effectively manage such demands, nurse managers are required to demonstrate heightened leadership, communication, decision‐making, and strategic planning capacities.

Nursing managers play a crucial role in the development and implementation of O2O‐style IPNS [17, 18]. They are not only responsible for organizing and coordinating O2O‐style IPNS but also for improving its operational efficiency and expanding its reach. Managerial decisions on nurse deployment, service scheduling, and risk control directly influence the quality, efficiency, and long‐term viability of O2O‐style IPNS [19]. Maintaining an appropriate balance between service demands and staff capacity is essential to safeguarding nurses’ physical and mental well‐being [20]. At the same time, nursing managers are central to safeguarding care quality and patient safety throughout the delivery of O2O‐style IPNS [21]. Given the offline and decentralized nature of IPNS, nursing managers must establish rigorous quality control systems and formulate service protocols that address core procedures such as nurse dispatching, on‐site care, and reporting, based on both institutional standards and frontline practice [22]. Given the solitary and home‐based nature of O2O‐style IPNS, nursing managers must implement tailored safety measures and emergency plans to protect both nurses and patients from potential risks [23]. These measures include preservice risk assessment, GPS‐based nurse tracking, one‐touch emergency alerts, emergency contact protocols, and contingency plans for service failure. By monitoring order processing, gathering feedback from frontline nurses and service recipients, and reviewing incident reports, nursing managers can detect inefficiencies—such as delayed service delivery, poor nurse–patient matching, or inconsistent documentation. Drawing on their practical experience, they translate these observations into concrete adjustments to scheduling rules, training content, and platform protocols, thereby continuously refining the O2O‐style IPNS model [24, 25]. At the same time, because O2O‐style IPNS operates across organizational boundaries, its implementation also involves platform‐side management functions such as order processing, user communication, operational coordination, and technical support. Thus, the management of IPNS extends beyond hospital nursing management and involves multiple interconnected managerial functions across healthcare institutions and service platforms.

Currently, managers involved in this emerging service model must navigate a range of challenges associated with workforce allocation, nurse safety, service quality control, interdepartmental coordination, platform–hospital collaboration, and sustainable operation. Existing research on IPNS has largely focused on nurses’ experiences, service design, patient needs, and quality assurance, with relatively limited attention to the experiences of those responsible for managing and coordinating its implementation across different organizational settings. This gap limits our understanding of how O2O‐style IPNS is organized and managed in real‐world practice. Therefore, this study aimed to describe the experiences of managers directly involved in the implementation and management of O2O‐style IPNS in China. Specifically, the study addressed two research questions: (1) What are managers’ experiences of implementing and coordinating O2O‐style IPNS? and (2) How can managers be supported in addressing challenges related to workforce management, service delivery, and interorganizational coordination?

## 2. Methods

### 2.1. Design

A descriptive qualitative design was used to describe managers’ experiences of implementing and managing O2O‐style IPNS. The findings of this study were presented in alignment with the Consolidated Criteria for Reporting Qualitative Research (COREQ) [26]; see Appendix 1 for a detailed outline of this alignment.

### 2.2. Participants and Settings

This study used maximum variation purposive sampling to recruit managers involved in O2O‐style IPNS from healthcare institutions and service platforms across different regions of China. A total of 11 study sites were included, comprising 8 hospitals and 3 service‐platform organizations across 8 cities. The sites varied in organizational type, geographic location, service volume, and implementation maturity. Monthly service volumes ranged from approximately 30 to over 400 orders, and implementation histories ranged from 6 months to over 3 years. This variation enabled the study to capture managerial experiences across diverse O2O‐style IPNS implementation contexts.

Participants were eligible if they (1) had been directly involved in the management or coordination of O2O‐style IPNS for at least 6 months; and (2) held substantive responsibility for one or more components of IPNS implementation or service management, including service quality management, nurse coordination and scheduling, order processing and delivery, platform operations, hospital–platform coordination, service promotion, or technical/operational coordination related to service delivery. Participants could be affiliated with either healthcare institutions or service platforms. Professional background was not used as an eligibility criterion because the study focused on managerial experiences related to IPNS implementation rather than experiences specific to a single profession. Individuals whose involvement was limited to technical or administrative support without substantive responsibility for IPNS management, coordination, or implementation were excluded.

At the participant level, variation was further sought in managerial responsibility and duration of IPNS management experience. Participants were not recruited according to predetermined strata; rather, their different roles reflected the multiple interconnected managerial functions involved in O2O‐style IPNS.

With institutional approval obtained, we contacted the chief nursing officers of selected hospitals to introduce the study’s purpose and procedures. With their support, we were connected to eligible hospital‐based nurse managers and platform managers. Potential participants were identified based on their direct involvement in IPNS management and their ability to contribute variation in managerial responsibility, institutional affiliation, implementation experience, and service context. Each potential participant was then approached individually via WeChat or mobile phone. Because recruitment was conducted purposively rather than from a predefined finite sampling frame, a single numerical size of the accessible population was not established. All invited managers agreed to participate, with no refusals or withdrawals.

### 2.3. Data Collection

The data collection was conducted through semistructured interviews. The research team predetermined the interview outline, mainly focusing on understanding the experiences of IPNS managers. Key questions included the following:

i. How would you describe your understanding of IPNS and your role in its implementation?
ii. Can you share any meaningful or memorable experiences you have had while managing IPNS, including either routine situations or special cases?
iii. What specific challenges or difficulties have you encountered in managing or delivering IPNS, and how did you respond to them?
iv. What kinds of support or resources would help you better manage IPNS in your current setting?
v. Based on your experience, what suggestions do you have for improving the implementation or day‐to‐day operation of IPNS?

Probing questions were used as needed to encourage elaboration. The guide was applied flexibly, allowing interviewers to adapt according to the participant’s responses and emerging topics.

First, the first author, a female nurse researcher with doctoral‐level training, conducted a pilot interview with a nursing manager at Beijing YouAn Hospital. The first author had received systematic training in qualitative research and possessed a strong research foundation in IPNS. To reduce potential bias arising from the researcher’s prior understanding of IPNS, reflective notes were maintained throughout the data collection and analysis process.

The interviews were conducted between March and June 2024, either face‐to‐face at the participants’ workplace or remotely via Tencent Meeting, depending on their availability and preference. Tencent Meeting, a widely used professional platform in China, was familiar to all participants and regularly used in their daily work, ensuring smooth participation in the interviews without technical barriers. The time and setting of each interview were arranged individually to minimize disruption to participants’ routine work. All interviews were conducted in a one‐on‐one format to ensure privacy and encourage open sharing. Informed consent was obtained from all participants, either in written form (for in‐person interviews) or oral form (for online interviews), in accordance with the study’s ethics approval. All participants were also informed that the interviews would be audio‐recorded for research purposes. No repeat interviews were conducted, as rapport between the researcher and participants was well established and each interview yielded sufficiently rich data [26]. This approach is also consistent with how semistructured interviews are most commonly conducted [27]. In this study, qualitative data analysis was carried out concurrently with data collection. When no new categories or themes emerged during the ongoing analysis, it indicated that saturation had been reached, and data collection was therefore discontinued [28]. Given that participants came from both healthcare institutions and service platforms, the research team also attended to whether role‐related perspectives were sufficiently represented in the developing thematic framework. Site variation was also considered throughout recruitment and data collection to ensure that experiences from different organizational and implementation contexts were captured.

A total of 24 participants were recruited from 11 study sites across 8 cities in China, including 18 hospital‐based nurse managers from 8 hospitals and 6 platform managers from 3 service‐platform organizations. The inclusion of managers from both healthcare institutions and service platforms was intentional because O2O‐style IPNS involves multiple interconnected managerial functions across the service pathway. Hospital‐based participants primarily contributed perspectives related to nursing workforce management, service quality, and institutional coordination, whereas platform managers contributed perspectives related to service operations, order management and delivery, user engagement, platform–hospital coordination, and related operational or technical management. Of the six platform managers, five had a nursing background and one had a non‐nursing professional background; all six held substantive managerial responsibilities directly related to IPNS implementation and service delivery. A demographic survey was also completed by participants, which included information such as gender, age, position, highest level of education, affiliated institution, and duration of management experience. Interview duration was recorded for each session, and descriptive statistics (mean and standard deviation) were calculated to summarize the length of the interviews. Each interview lasted approximately 47.02 min on average (SD = 10.35).

### 2.4. Data Analysis

The first author used Tencent Meeting’s built‐in transcription and audio‐recording features to generate the transcript and repeatedly listened to the audio recordings to verify accuracy and ensure fidelity to the original language, without adding commentary [29]. The transcribed documents were sent individually to the respective participants for member checking, allowing them to review and confirm the accuracy of the content [30]. Data were analyzed using a conventional content analysis approach as outlined by Hsieh and Shannon [29], which is appropriate when prior research or theoretical frameworks are limited. First, the first and second authors independently read the interview transcripts multiple times to gain immersion and an overall sense of the data [31]. Then, they reviewed the text line by line to identify significant words and phrases that captured key thoughts and concepts [32]. Initial codes were generated based on these highlighted units. Similar codes were grouped into categories based on conceptual similarity, and these categories were further abstracted into broader subthemes and overarching themes [29]. The coding structure was iteratively refined through discussion among all authors until consensus was reached.

Participants’ managerial roles and institutional affiliations were treated as contextual characteristics rather than predetermined analytic strata. The primary analysis focused on shared experiences of implementing and managing O2O‐style IPNS across the full dataset. During category refinement and interpretation, the research team also considered whether particular perspectives were shared across different managerial roles or were more strongly associated with specific service functions or institutional settings.

### 2.5. Trustworthiness

Credibility was enhanced through transcript verification, member checking, concurrent data collection and analysis, and reflective note‐taking. Transferability was supported by maximum variation sampling and detailed reporting of the study context and participant characteristics. Dependability was strengthened through systematic data analysis and iterative refinement of codes, categories, and themes through research‐team discussion and consensus. Applicability was supported by contextualized findings and quotations from participants with different managerial roles and institutional affiliations, enabling readers to judge their relevance to comparable settings.

### 2.6. Ethical Considerations

This study was approved by the Medical Ethics Committee of Capital Medical University (Z2022SY029). Given the interviewer’s previous work and research in the field, many participants were previously acquainted with the researcher. To avoid any perception of coercion, the voluntary nature of participation was explicitly emphasized, and participants were assured that their decision would not impact their professional standing. Furthermore, participants were informed of their right to withdraw from the study at any time without providing a reason. All data were anonymized and stored securely to protect participants’ privacy and confidentiality.

## 3. Results

The final sample comprised 24 participants from 11 study sites across 8 cities in China, including 18 hospital‐based nurse managers from 8 hospitals and 6 platform managers from 3 service‐platform organizations. The average interview duration was 47.02 min (SD = 10.35). Their characteristics are shown in Table 1. Notably, Participants N19 and N24 had longer IPNS‐related management experience because their institutions were involved in early local O2O‐style home nursing service practices in Ningbo before the national pilot policy for Internet + Nursing Service was issued in 2019. Therefore, their management experience was calculated based on involvement in both earlier local practices and the official Internet + Nursing Service pilot. In particular, N24 was a platform‐based participant whose experience referred to postgraduation involvement in order delivery management and service coordination on the platform, rather than formal hospital‐based nursing management.

**TABLE 1:** Characteristics of participants.

Code	Sex	Age	Position/role	Education	Affiliation	IPNS‐related management experience (months)
N1	Female	45	Head nurse	Bachelor	Hospital	36
N2	Female	43	Head nurse	Bachelor	Hospital	36
N3	Female	46	Chief nursing officer	Bachelor	Hospital	10
N4	Female	48	Head nurse	Associate degree	Hospital	12
N5	Female	46	Associate chief nursing officer	Bachelor	Hospital	36
N6	Female	48	Chief nursing officer	Bachelor	Hospital	26
N7	Female	46	Associate chief nursing officer	Bachelor	Hospital	6
N8	Female	36	Head nurse	Bachelor	Hospital	36
N9	Female	42	Registered nurse, platform manager	Bachelor	Platform	48
N10	Female	54	Associate chief nursing officer	Bachelor	Hospital	24
N11	Female	39	Head nurse	Bachelor	Hospital	18
N12	Female	40	Registered nurse, platform manager	Bachelor	Platform	22
N13	Female	34	Head nurse, platform manager	Bachelor	Platform	23
N14	Female	37	Head nurse	Bachelor	Hospital	12
N15	Female	36	Head nurse	Bachelor	Hospital	36
N16	Female	50	Chief nursing officer	Bachelor	Hospital	46
N17	Female	57	Chief nursing officer	Bachelor	Hospital	56
N18	Female	49	Chief nursing officer	Master	Hospital	34
N19	Female	57	Chief nursing officer	Bachelor	Hospital	96
N20	Female	46	Associate chief nursing officer	Bachelor	Hospital	12
N21	Female	52	Chief nursing officer	Bachelor	Hospital	48
N22	Male	42	Platform manager	Associate degree	Platform	50
N23	Female	40	Head nurse, platform manager	Master	Platform	36
N24	Female	29	Registered nurse, platform manager	Bachelor	Platform	72

Four interrelated themes were inductively developed from the data, reflecting how managers understood the value of IPNS, mobilized resources, navigated organizational boundaries, and considered the conditions necessary for sustainable implementation. These themes are illustrated in Figure 1.

**FIGURE 1:**
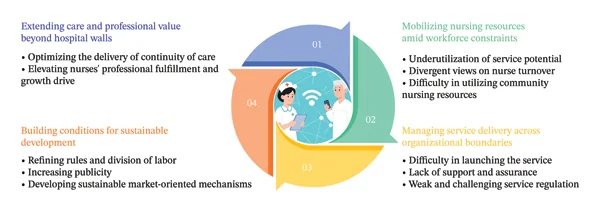
Thematic framework.

To make the contribution of different managerial perspectives transparent, participant quotations are identified by participant code, managerial role, and institutional affiliation. Role‐related similarities and differences are highlighted in the findings where they were evident in the data.

### 3.1. Extending Care and Professional Value Beyond Hospital Walls

#### 3.1.1. Optimizing the Delivery of Continuity of Care

Conventional continuity of care in hospital settings has been described as primarily relying on discharge education and follow‐up phone calls. These approaches were considered limited in scope and insufficient for meeting patients’ ongoing care needs. Staff shortages in hospitals often made it difficult for managers to provide in‐depth, postdischarge guidance. The emergence of IPNS created new opportunities for delivering in‐home care to patients who met specific conditions. It enabled nurses to enter patients’ home environments, provide face‐to‐face instructions for wound care and rehabilitation exercises, and monitor recovery progress over time. Managers widely acknowledged that this model meaningfully enhanced the effectiveness of continuity of care.

*To be honest, we didn’t do a great job with continuity of care before. It was mostly just some discharge instructions and a few phone calls—how much can that really help? Patients were anxious, and so were we, but the hospital was so busy we couldn’t spare anyone to go deeper. (N3, Chief nursing officer, hospital)*

*Patients who need and can afford it can place orders, and we’re able to go to their homes, teach them one-on-one how to care for their wounds or do rehab. We can also spot what they’re doing wrong and correct it. When we go back after a while and see real progress, it’s really rewarding. This service made all the difference. (N8, Head nurse, hospital)*

*Post-discharge care is still far from sufficient. Every patient who has just left the hospital needs someone to explain what to do next and provide ongoing guidance and support. We usually develop a care plan based on the patient’s needs, which may cover a few days, a week, or even a month. (N13, Head nurse/platform manager, platform)*

#### 3.1.2. Elevating Nurses’ Professional Fulfillment and Growth Drive

Managers generally believed that IPNS not only expanded the scope of service delivery but also created more opportunities for nurses to demonstrate their professional skills and gain patient recognition. Some nurses reported a strong sense of achievement during service delivery, expressing a feeling of being “needed.” Meanwhile, patients’ ongoing demand for high‐quality care and healthy competition among peers subtly fostered nurses’ motivation for continuous learning and professional development. This combination of internal and external incentives further promoted active nurse participation and contributed to the overall improvement of service quality.

*There’s a nurse in our department who’s really motivated—she has already taken more than 80 orders. She often tells me how much she feels her skills are truly helping patients and solving real problems. That sense of being needed gives her a great deal of fulfillment. (N7, Associate chief nursing officer, hospital)*

*Some patients stick to the same nurse after a good experience, and that creates a kind of positive competition—everyone wants to deliver the best service. Nurses start actively learning and improving their skills because they don’t want to be caught off guard by patient questions. For younger nurses who haven’t qualified to take orders yet, it’s also motivating. Seeing their seniors gain recognition pushes them to catch up. (N1, Head nurse, hospital)*

*In the hospital, care is often physician-centered, and much of what nurses do—especially patient education—is easily overlooked. But in the home, patients really need that guidance. Nurses can see much more clearly how their knowledge and skills help patients, and they feel that their professional value is truly being recognized. (N9, Registered nurse/platform manager, platform)*

### 3.2. Mobilizing Nursing Resources Amid Workforce Constraints

#### 3.2.1. Underutilization of Service Potential

A key barrier to scaling up O2O‐style IPNS was not a lack of qualified personnel but the underutilization of available nursing capacity, reflecting deeper motivational deficits rooted in organizational and systemic factors. Despite being eligible and enrolled on the platform, many nurses refrained from taking service orders—revealing that formal qualifications alone do not translate into behavioral engagement, particularly when institutional incentives and perceived value are misaligned. Motivation was shaped by a constellation of influences, including insufficient financial rewards, unclear career progression pathways, and the absence of recognition mechanisms for work conducted outside traditional clinical settings. The engagement of frontline nurse leaders proved especially pivotal, as their own valuation of IPNS shaped whether they positioned it as a meaningful professional development activity or a disruptive task. Interviewees emphasized that participation could not be mandated; instead, managers needed to implement structural incentives—such as promotion‐linked credits, departmental performance rewards, and public recognition—to integrate IPNS meaningfully into nurses’ professional identities.

*Currently, there is the problem of insufficient manpower to take orders; orders are sent to our hospitals, but no one can or is willing to go. There are actually a lot of nurses who are qualified to serve or who are registered on the platform, but they are not motivated. (N2, Head nurse, hospital)*

*When we initially started, the nurses were highly motivated, and after taking orders for a few times, there was nothing more to do. We rejoined the new members, and the first thing we should do in our work is to care more about the nurses. The second thing is that there should be a lot of incentives and rewards, such as in the area of promotion and title. By adding these, the nurses will definitely be more motivated… to mobilize the motivation of the head nurse, for example, at the end of the year, the department that participates in the most IPNS, the head nurse and the whole team have rewards and so on. (N5, Associate chief nursing officer, hospital)*

*If nurses themselves are not motivated, the hospital cannot simply force them to take orders. Participation is voluntary, so there is only so much the hospital can do. (N24, Registered nurse/platform manager, platform)*

#### 3.2.2. Divergent Views on Nurse Turnover

A clear role‐related difference emerged regarding the perceived impact of IPNS on nurses’ employment stability. Hospital‐based nurse managers tended to view IPNS as a potential threat to workforce retention and clinical commitment, whereas platform managers generally regarded this risk as limited. Hospital‐based nurse managers worried that nurses might reduce their commitment to clinical work or even consider leaving their jobs due to the additional income they could earn from participating in these services. There was also concern that the involvement of external capital might lead to a depletion of professional nursing resources, potentially impacting hospital operations. In contrast, platform managers tended to regard this risk as limited. From their perspective, full‐time participation in IPNS was unlikely to replace hospital employment because it lacked the job security, social insurance, and organizational stability associated with formal hospital positions. This contrast revealed a role‐based tension between hospitals’ concern about protecting nursing resources and platforms’ emphasis on the practical constraints of independent IPNS work.

*We are less active in this. One reason is that when a nurse has extra income, their mind may not be on clinical work. In addition, our nurses are all professionals. If community elderly institutions or private hospitals require professional nurses, our nurses, as readily available resources, could be poached with the involvement of external capital, which could impact our hospital’s operations. This would be a case of losing something significant for a small gain. (N6, Chief nursing officer, hospital)*

*But actually, there is no impact on stability. It is possible for nurses to leave hospitals because of low wages and move to other well-paying careers, but working full-time in IPNS is unlikely due to the lack of health insurance and stability. (N9, Registered nurse/platform manager, platform)*

#### 3.2.3. Difficulty in Utilizing Community Nursing Resources

In the implementation of O2O‐style IPNS, community hospital nurses are geographically well positioned and theoretically responsible for home visits, primarily involving health education and chronic disease follow‐up. This suggests that they could help address nursing workforce shortages in home‐based services. However, their limited exposure to technical procedures and lack of inpatient experience make it difficult for them to meet the clinical demands of IPNS, especially for more complex or urgent nursing tasks. Community hospitals often lack practice opportunities and a sufficient number of cases, leading to a gap in the technical skills and experience of their nurses compared to those in general hospitals. Without in‐patients, the frequency of nursing procedures in community hospitals is low, making it challenging for their nurses to attain the same level of technical proficiency as nurses working in general hospitals. This discrepancy in skill levels limits the role of community hospitals in IPNS and hinders their ability to handle more nursing service orders. Concerns about the readiness of community nurses were shared across institutional roles, with both platform‐based and hospital‐based managers emphasizing limitations in clinical exposure and emergency management. This situation presents a challenge for nursing managers, who must decide whether to involve community nurses in IPNS delivery, and, if so, how to provide additional training and supervision to ensure service quality.

*There is no way for community hospitals to take up orders because nursing is a skilled job, and practice makes perfect. In China, the workload of nurses in community hospitals is much lower than that in general hospitals, and the number of patients they care for is also significantly smaller. For example, with injections, nurses in hospitals give people infusions every day, so they are naturally skilled. However, nurses in community hospitals do not receive a similar workload. And community hospitals don’t have people hospitalized, and there are no difficult cases. The ability of community hospital nurses is definitely not as strong as that of general hospitals. (N24, Registered nurse/platform manager, platform)*

*Community hospital nurses aren’t really good at handling emergencies. If something serious happens during the service, I don’t think they’d know what to do — and most patients feel the same way. (N16, Chief nursing officer, hospital)*

*In the township areas, we simply do not have enough nurses with specialist skills. If we want to provide comprehensive and professional home nursing care, we need to train more nurses and strengthen their clinical and emergency-response skills. (N7, Associate chief nursing officer, hospital)*

### 3.3. Managing Service Delivery Across Organizational Boundaries

#### 3.3.1. Difficulty in Launching the Service

While national policies strongly endorse IPNS development, their implementation at the ground level is often stalled by fragmented provincial regulations and opaque administrative pathways, which transform encouragement into hesitation. The resulting administrative burden, coupled with policy ambiguity, fosters institutional inertia—hospitals comply passively rather than pursue active promotion. Additionally, concerns from hospital management hinder a smooth launch. Consequently, the service struggles to achieve the rapid growth expected during its start‐up phase, which in turn affects its broader rollout. However, some interviewees noted that once the service is able to operate smoothly and enter a regular management phase, subsequent operations can become automated, significantly reducing the management burden.

*There are a lot of objective reasons why the service hasn’t grown exponentially. Hospitals are under pressure to do it, but they have their own concerns. Hospitals don’t actively promote the service; they simply provide basic information on their website, resulting in poor outreach to patients. (N22, Platform manager, platform)*

*On the hospital side, we had a hard time convincing hospital leaders to push for this activity. The national policy encourages hospitals to participate, but there are all sorts of regulations at the provincial level. Providing materials for reporting means hospitals have to go through a set of processes, and since they don’t make a profit, they simply don’t think it’s a good idea. (N23, Head nurse/platform manager, platform)*

*But after running it, everyone is only divided into their own small part of the task. There’s no extra management required; it’s all normalized. (N8, Head nurse, hospital)*

#### 3.3.2. Lack of Support and Assurance

The promotion of IPNS faces significant challenges due to a lack of support and assurance. Many hospitals and their leadership were primarily motivated to implement these services by policy requirements, typically driven by government initiatives, rather than by an intrinsic desire to innovate. While policy can act as a catalyst, hospitals are often hesitant to embrace potential negative outcomes, especially when lacking the financial incentives to invest. The attitude of hospital leadership is crucial to service promotion; their level of support directly influences the ease or difficulty of implementing these services within nursing departments and units. Some interviewees noted the difficulty of promoting the service solely under the banner of the nursing department or unit. The absence of unified publicity and promotional efforts within the hospital further hampers the initiative’s reach. Additionally, hospitals are reluctant to shoulder the risks associated with quality and safety issues due to the fear that “more work leads to more mistakes.”

*It’s difficult for us. It’s challenging to push this forward in the name of the nursing department and the unit… The stance of hospital leadership on service development is very important. (N14, Head nurse, hospital)*

*Hospitals have no revenue from this. Doing it can only be driven by policy, but hospitals are not willing to bear the consequences of poor service quality and are afraid of doing more and being wrong … I feel caught between a rock and a hard place. An external service like this leaves me feeling powerless when dealing with both subordinates and superiors, diminishing my motivation to promote it. (N18, Chief nursing officer, hospital)*

*From the platform side, working with hospitals is actually quite difficult. We often need support from the government to move things forward. Hospitals tend to be cautious because they worry that the more they do, the more likely something is to go wrong. (N24, Registered nurse/platform manager, platform)*

#### 3.3.3. Weak and Challenging Service Regulation

O2O‐style IPNS faces significant challenges in regulation, making it difficult to achieve the same level of oversight as clinical nursing. Difficulties in regulating offline service quality were reported across managerial roles. Both platform‐based and hospital‐based managers described limited visibility into what occurred during home visits and the inadequacy of existing monitoring mechanisms. Existing tools are inadequate for effectively monitoring the offline service process of nurses, resulting in difficulties in ensuring service quality. The core issue lies in the inability to capture the details of nurses’ work in patients’ homes, such as timely communication with clients, the accuracy of their assessments, the content of their communications, and any special circumstances that arise on‐site. Currently, there is a lack of effective supervision of these key aspects, relying solely on postservice feedback. Without a standardized service record and regulatory mechanism, quality assessment becomes more arbitrary. Additionally, current regulatory methods, like telephone callbacks, are lagging; feedback is often collected long after the service has concluded, impacting the timeliness and effectiveness of regulation.

*The supervision of offline service quality is more difficult than clinical work, and our current tools are insufficient to effectively support the supervision of nurses. There is no way to monitor whether the nurse has called the client, whether they have done the assessment, and whether the assessment is accurate. What communication did they actually have with the patient? Were there any special circumstances that occurred on-site? We have no way of knowing any of this. We can only make comprehensive judgments through feedback from the nurse and the client. (N12, Registered nurse/platform manager, platform)*

*The monitoring of quality is mainly in the form of telephone callbacks, but this is lagging. Maybe 20 days after the end of the service, we call and ask for feedback, which is actually not very meaningful. (N15, Head nurse, hospital)*

*At the moment, most of our supervision takes place after the service, mainly through follow-up phone calls. The documentation is not always standardized, and there is very little real-time oversight in the background. If this service is going to be managed properly, there really needs to be dedicated staff responsible for monitoring and follow-up. (N20, Associate chief nursing officer, hospital)*

### 3.4. Building Conditions for Sustainable Development

#### 3.4.1. Refining Rules and Division of Labor

Interviewees highlighted the risks associated with the lack of detailed rules and systems for O2O‐style IPNS. For instance, while nurses are prohibited from adding patients on WeChat privately, this behavior is often tacitly permitted because patients prefer consulting through WeChat. This poses risks such as taking private orders and other safety hazards. To ensure service quality, clear regulations are needed to restrict services that fall outside the scope of the order and to appropriately handle needs unrelated to nursing. Additionally, standardizing procedures for waste disposal, service etiquette, and emergency plans is necessary to maintain the professionalism and safety of the services.

*Currently, so many details are not specified in the rules. Let me give you an example: nurses will privately add patients’ WeChat. We repeatedly stress that this is not allowed, but we can only acquiesce because so many patients come to consult nurses through WeChat. We can only emphasize that nurses are not allowed to take orders privately. (N20, Associate chief nursing officer, hospital)*

To promote the development of IPNS, respondents expressed a desire for clearer cooperation between platforms and hospitals, with a more defined division of responsibilities to enhance the professionalism of the services. Currently, the roles of platforms and hospitals in service delivery are unclear, leading to potential pitfalls. Interviewees suggested that platforms should take charge of technical support, process management, and the initial auditing of nursing services, while hospitals should focus on auditing nurses’ qualifications and providing nursing staff to ensure their professional competence. Additionally, once patients are discharged from the hospital and use nursing services, they should not directly contact the hospital since this is considered an out‐of‐hospital service. To ensure quality and safety, platforms and hospitals should establish a joint quality control mechanism to clarify who is responsible for coordinating and resolving issues between nurses and patients, providing timely support when needed.

*Now the division of labor between the platform and the hospital is not clear, and this hidden danger can be too much. The platform should provide technical protection and audit, and the hospital should audit nurse qualifications and provide personnel. Patients discharged from the hospital should not contact the hospital again because this is an out-of-hospital service. When nurses or patients need to coordinate and solve problems, it should be clearly defined who they should contact. (N1, Head nurse, hospital)*

*Hospitals may say, ‘This happens outside the hospital, so it has nothing to do with us—the platform should be responsible.’ But the platform cannot manage the nurses on its own; hospitals still need to take responsibility for overseeing their qualifications and professional practice. (N24, Registered nurse/platform manager, platform)*

#### 3.4.2. Increasing Publicity

Respondents expressed the need for greater publicity of O2O‐style IPNS to raise awareness and utilization. Currently, many patients are unaware of this service or the specific care support they can receive through it. Even if patients have a need, they might not realize that they can utilize IPNS. Service providers should actively identify potential patient needs and use effective publicity to increase public awareness of the service. Respondents suggested that IPNS publicity should be as impactful as that for food delivery and ride‐hailing services, where orders can be placed online and clients simply wait at home, so that the public readily considers and utilizes the service as needed, thereby promoting its widespread adoption.

*Nurses usually only mention the service when they’re sure a patient needs it, so most patients aren’t even aware it exists. We’ve put flyers at the nurses’ station, but hardly anyone picks them up. (N9, Registered nurse/platform manager, platform)*

*At present, insufficient publicity is a major barrier. Patients don’t know that this type of service exists or what services nurses can provide for them. Even if they have a need, they don’t realize it. The key is to tap into the patients’ needs. At the very least, people should know about this service the way they know about Meituan and Didi (the most common food delivery and ride-hailing apps in China)—so they understand it and know that when they need it, they can access it. (N6, Chief nursing officer, hospital)*

*Publicity was quite strong at the beginning, but it gradually faded over time. Most patients still do not know that this service exists, so I think hospitals need to continue promoting it. (N14, Head nurse, hospital)*

#### 3.4.3. Developing Sustainable Market‐Oriented Mechanisms

Interviewees believe that developing market‐oriented mechanisms for O2O‐style IPNS was important for ensuring long‐term sustainability. They noted that it would be difficult for the government to fund the service indefinitely. A more sustainable payment and service operation mechanism could help provide nurses with appropriate compensation and improve patients’ service experience. By leveraging market mechanisms, service resources can be allocated more rationally, supply and demand can be better balanced, and overall service quality can be improved. Additionally, while government subsidies primarily focus on covering the disabled population, market‐oriented mechanisms could help respond to the diverse needs of other patient groups and support the sustainable development of the service.

*Commercialization of the service is an inevitable trend and a requirement for long-term development. The government cannot afford to pay for this service. Only through commercialization can nurses receive satisfactory income, and patients have a better experience… The group of bedridden and disabled individuals that the government wants to subsidize is already covered by long-term care insurance. (N9, Registered nurse/platform manager, platform)*

*If all the services were covered by insurance, a lot of people who could’ve just come to the hospital would start placing orders too. The number of orders might go beyond what we can handle. (N16, Chief nursing officer, hospital)*

*You cannot sustain this service on goodwill alone. The price has to be acceptable to patients and their families, while also being fair enough for nurses. Only then can the service continue in a sustainable way. (N23, Head nurse/platform manager, platform)*

## 4. Discussion

Previous studies have shown that home‐based and context‐specific nursing care can improve patients’ self‐efficacy and satisfaction by bringing professional support closer to patients’ daily lives [33, 34]. Our findings extend this evidence by showing that, in O2O‐style IPNS, continuity of care is not only a matter of postdischarge education or remote follow‐up but also depends on whether hospitals can organize nurses, platforms, and service processes to support care delivery beyond hospital walls. However, unlike systems where home health services are institutionally embedded, Chinese hospitals still face structural fragmentation, making such continuity difficult to achieve. This finding highlights that the development of O2O‐style IPNS requires not only clinical service extension but also stronger organizational coordination and managerial support. In addition, previous research has emphasized the importance of professional motivation in sustaining nurses’ participation [35]. Consistent with this view, our findings suggest that nurses’ motivation in O2O‐style IPNS may be strengthened not only through external rewards but also through visible patient improvement, professional recognition, and opportunities for personal growth.

Additionally, the findings indicate that managers are particularly concerned about the adequacy of the nursing workforce, which emerged as the primary challenge in managing their subordinates. Similar to previous studies, although nurses demonstrate a relatively high willingness to participate, the actual participation rate remains low due to factors such as relevant policies, laws and regulations, role conflict, and healthcare safety guarantees [36, 37]. For example, a report from Nanning Cloud Hospital revealed that only 967 out of 3661 registered nurses (26.41%) were actively participating in O2O‐style IPNS, which provides empirical support for the observed gap between registration and actual engagement [38]. Therefore, the managers face a situation of insufficient availability of nurses. They place their hopes on motivating nurses by enhancing salary, implementing performance incentives, and offering career advancement opportunities. However, previous research indicates that not all of these factors impact nurses’ motivation [39, 40]. Therefore, as required by policy [4], tailored measures should be developed based on local conditions to effectively supplement the nursing workforce.

Hospital‐based nurse managers expressed concern that participation in IPNS might affect workforce retention and clinical commitment, whereas platform managers generally considered this risk to be limited. Previous studies have not established a direct association between part‐time work and nurse turnover; rather, turnover intention has been associated with unfair work allocation, dissatisfaction with salary, and work‐related fatigue [41, 42]. Therefore, flexible scheduling and equitable workload allocation may be particularly important when nurses undertake IPNS in addition to their regular clinical duties.

Contrary to previous studies that encouraged community hospital nurses to participate in providing IPNS, our findings indicate that these nurses face limited practical opportunities due to lower patient volume. This discrepancy likely arises from differences in healthcare settings and the alignment between supply and demand. Previous studies [42, 43] emphasized adequate nurse staffing by supporting factors such as the geographical accessibility of community health centers, the high workload of tertiary hospital nurses (limiting their availability for these services) [44], and the strong rapport that community nurses build with local patients, alongside their familiarity with managing chronic conditions, which supports home visits [45]. However, managers in this study prioritized maintaining service quality and addressing potential delivery risks. When providing offline services in patients’ homes, due to the lack of team support and emergency equipment, managers must carefully evaluate nurses’ qualifications. Consequently, future research should focus on exploring the specific challenges community nurses encounter in delivering IPNS, as well as how these challenges influence nursing practices and patient outcomes in home settings [46].

The approval process for IPNS is often complex and lengthy, adding challenges to its implementation. Currently, the implementation procedures vary across regions due to differing provincial‐level requirements, with local health authorities responsible for authorization. While the criteria for nurse, institution, and platform participation are still being clarified—with some variation in procedures and regulatory frameworks—these reflect the early stages of policy development [47]. The lack of unified laws, standardized approval mechanisms, and consistent reimbursement policies further impedes scale‐up. Nevertheless, a recent survey of nursing managers highlighted key areas for improvement, including enhanced governmental support and better‐developed payment systems, which are gradually being addressed to support the broader standardization and expansion of IPNS [48]. Additionally, participants in our study were also concerned about insufficient support from hospital executives, who may be hesitant due to potential liability in case of adverse outcomes. This is not entirely in line with previous studies. Prior research mainly explored the experiences of nurses involved in services, with an emphasis on the lack of peer support, multidisciplinary team collaboration, and policy backing [12]. Participants, particularly hospital‐based nurse managers, emphasized that the attitude of senior hospital leadership plays a crucial role in the effective implementation of services. Additionally, although managers are committed to maintaining quality and ensuring the smooth delivery of services, their work is hindered by the high costs of appropriate monitoring tools and limited monitoring capabilities due to stringent privacy policies [49, 50]. Our study indicates that these three aspects—approval processes, executive support, and regulatory tools—exceed the control and resources of nursing managers, creating barriers to motivating and advancing this service effectively. However, while the initial implementation is hindered by procedural and institutional barriers, once the system is in place, routine operations proceed smoothly with fewer management demands. The current preservice review process involves dual‐level screening by both the platform and the hospital, primarily assessing whether the patient’s health condition is appropriate for home‐based nursing care [51]. Following order acceptance, services are consistently completed as planned, with very high satisfaction levels reported and adverse events or complaints rarely reported, as documented in previous studies [52].

Our study also explored the expectations of managers for the service. Managers have a strong desire for clear guidelines and distinct duty assignments, which aligns with previous studies [50, 53]. The involvement of multiple institutions and changes in service locations introduce many uncertainties to the smooth delivery of services. Their primary motivation is to ensure smooth service operation. At the policy level, health authorities should clarify the regulatory boundaries, service standards, and accountability mechanisms for O2O‐style IPNS. At the institutional level, hospitals should define internal responsibilities for nurse dispatching, risk assessment, quality monitoring, emergency response, and interdepartmental coordination. Service platforms should further clarify their responsibilities in order management, information tracking, user communication, and technical support. Clear responsibilities are a prerequisite for accountability, which helps prevent disputes between departments and institutions due to ambiguous role delineation, thereby supporting service quality.

Another key expectation expressed by participants was the need to strengthen promotion of the service model. This finding aligns with previous research, which suggests that the development of O2O‐style IPNS requires expanding the intensity and scope of its promotion [54, 55]. Additionally, our study uncovered new insights, such as identifying and uncovering user needs. This may be due to the inclusion of platform management personnel in our interviews. They indicated that many discharged patients are potential clients of IPNS, but they are either unaware of the service or do not realize they can access offline in‐home care. Identifying potential users can improve the effectiveness of promotional efforts.

In addition, participants expressed expectations for more sustainable market‐oriented mechanisms in O2O‐style IPNS. Previous studies have primarily emphasized integrating such services into medical insurance to improve affordability for those in need [54, 56]. In contrast, participants in our study raised concerns about the potential unintended consequences of full insurance coverage, including overutilization, inefficient resource allocation, and increased risks of fraud and inappropriate claims [57], particularly in the context of ongoing cost‐containment pressures within China’s expanding health insurance system [58]. These concerns may be particularly relevant because the out‐of‐pocket cost of IPNS is currently reported to be 5–10 times higher than that of comparable in‐hospital services covered by medical insurance, potentially increasing demand beyond the nursing system’s capacity [59]. At the same time, China’s long‐term care insurance already provides coverage for individuals with severe disabilities, helping to ensure essential nursing services for those most in need [60]. This suggests that public insurance and market‐oriented payment mechanisms may need to be balanced, with public programs protecting vulnerable populations and noninsurance payment mechanisms responding to more diverse service needs. In addition to these systemic concerns, many nurses expressed dissatisfaction with the current compensation system and often perceived in‐home care as a form of personal dedication rather than professional labor [61, 62]. If patients regard the service merely as a paid transaction, it may further diminish nurses’ motivation. Therefore, rather than emphasizing commercialization as profit seeking, a carefully regulated market‐oriented mechanism may help sustain O2O‐style IPNS by ensuring fair compensation, recognizing nurses’ professional labor, and improving patient experience. In terms of affordability, policy adjustments should be designed with the premise of ensuring equitable income for nurses [61]. Furthermore, service managers indicated that while they can express their needs regarding policy guidance, providing actionable insights to drive change remains a challenging and ongoing task. The themes and conclusions drawn from the analysis accurately reflect the patterns and insights observed in the data, underscoring the reliability of our findings.

### 4.1. Study Limitation

This study has some noteworthy limitations. First, the majority of participants were female, reflecting the female‐dominated nature of the IPNS field. Second, the data collection was concentrated within a specific time period, which may somewhat limit the ability to reflect the evolving trends of IPNS over time. Third, although platform managers were intentionally included to provide complementary perspectives on service operation, order management, user engagement, and platform–hospital coordination, they represented a smaller proportion of the sample than hospital‐based nurse managers. In addition, only one participant had a non‐nursing professional background. This participant was retained because of substantive responsibility for platform‐based IPNS management and coordination; however, the findings predominantly reflect the perspectives of managers with nursing backgrounds. Future studies could recruit a broader range of platform‐based and non‐nursing managers to further examine how professional and organizational backgrounds may shape IPNS management experiences.

## 5. Conclusion

This study provides an in‐depth description of the experiences of managers directly involved in implementing and managing O2O‐style IPNS. Managers highlighted the value of the service in improving continuity of care and enhancing nurses’ sense of professional achievement and motivation for growth. However, despite these recognized benefits, they encountered substantial challenges in practice, particularly in workforce coordination and service promotion. As a new service approach enabled by information technology, the O2O‐style IPNS model places managers in a complex operational environment. Their primary concern in managing staff centers on personnel shortages, while on the service side, they worry about insufficient institutional support and safeguards. This lack of systemic support diminishes their capacity to drive progress and places them in a difficult position—balancing competing needs, limited resources, and the demand to maintain service quality.

To address these obstacles and promote the healthy, orderly growth of the service, interviewees emphasized the importance of clearly defining roles and responsibilities among hospitals, platforms, and nurses to reduce confusion and legal risks. They also advocated for stronger promotional efforts both within hospitals and among the public to raise awareness and improve service utilization. In addition, they suggested adopting sustainable market‐oriented mechanisms, such as performance‐based incentives and flexible participation options, to motivate nurses to engage in the service. By strengthening institutional support, clarifying collaborative responsibilities, and recognizing the professional value of nurses’ work beyond hospital settings, O2O‐style IPNS has the potential to reshape nursing service delivery and expand the professional role of nurses in China’s evolving healthcare system.

## 6. Implications for Nursing Management

The implementation of O2O‐style IPNS expands nurse managers’ responsibilities beyond traditional roles, requiring coordination across hospitals, platforms, and home‐based care. Managers addressed nurse participation challenges through flexible scheduling, performance incentives, and departmental recognition, enhancing motivation and service reliability. Concerns about nurse turnover were mitigated by emphasizing IPNS as supplementary, safeguarding job security and career stability. Although community nurses were geographically ideal, limited clinical skills led managers to recommend targeted training and supervision. Initial resistance from organizational complexity was overcome through proactive leadership engagement, strategic collaboration with platforms, and clear role delineation. Quality assurance challenges in home care prompted real‐time monitoring and standardized documentation practices. Additionally, nurse managers advocated for clearer regulations, standardized licensing, and gradual commercialization.

## Funding

This research was funded by the National Natural Science Foundation of China (No. 72174130).

## Disclosure

All authors have read and approved the final version of the manuscript. The funder had no role in the study design, data collection, analysis, or interpretation.

## Ethics Statement

This study design was reviewed and approved by the Ethics Committee of Capital Medical University (Approval No.: Z2022SY029).

## Conflicts of Interest

The authors declare no conflicts of interest.

## Supporting Information

Additional supporting information can be found online in the Supporting Information section.

## Supporting information


**Supporting Information** Appendix 1: Detailed presentation of the study’s alignment with the Consolidated Criteria for Reporting Qualitative Research (COREQ). This file provides an in‐depth outline of how the study adhered to COREQ guidelines, supporting the transparency and rigor of the qualitative research findings.

## Data Availability

Data are available on request from the authors.
